# Knockdown of GABA_A_ alpha3 subunits on thalamic reticular neurons enhances deep sleep in mice

**DOI:** 10.1038/s41467-022-29852-x

**Published:** 2022-04-26

**Authors:** David S. Uygun, Chun Yang, Elena R. Tilli, Fumi Katsuki, Erik L. Hodges, James T. McKenna, James M. McNally, Ritchie E. Brown, Radhika Basheer

**Affiliations:** 1 VA Boston Healthcare System and Harvard Medical School, Dept. of Psychiatry, West Roxbury, MA 02132 USA; 2 Stonehill College, Department of Psychology, Easton, MA 02357 USA

**Keywords:** Slow-wave sleep, CRISPR-Cas9 genome editing

## Abstract

Identification of mechanisms which increase deep sleep could lead to novel treatments which promote the restorative effects of sleep. Here, we show that knockdown of the α3 GABA_A_-receptor subunit from parvalbumin neurons in the thalamic reticular nucleus using CRISPR-Cas9 gene editing increased the thalamocortical delta (1.5–4 Hz) oscillations which are implicated in many health-promoting effects of sleep. Inhibitory synaptic currents in thalamic reticular parvalbumin neurons were strongly reduced in vitro. Further analysis revealed that delta power in long NREM bouts prior to NREM-REM transitions was preferentially affected by deletion of α3 subunits. Our results identify a role for GABA_A_ receptors on thalamic reticular nucleus neurons and suggest antagonism of α3 subunits as a strategy to enhance delta activity during sleep.

## Abstract (web summary)

Uygun et al. show that deletion of GABA_A_ receptors from the thalamic reticular nucleus using CRISPR gene editing in mice boosts the delta waves, indicating a role for GABA_A_ receptors on thalamic reticular nucleus neurons in NREM sleep delta oscillations.

## Introduction

Sleep is vital for maintaining physical and mental well-being. In particular, thalamocortical delta (1.5–4 Hz) oscillations present in deep non-rapid-eye-movement (NREM) sleep are implicated in a wide range of processes beneficial to health including synaptic homeostasis, cellular energy regulation, clearance of toxic proteins, cognitive performance and mood^1–4^. Conversely, insomnia, traumatic brain injury, obstructive sleep apnea, and other brain disorders are associated with interrupted/fragmented sleep, reduced deep NREM sleep and decreased delta wave power^5–8^. Although hypnotic agents which potentiate the activity of GABA_A_ receptors promote sleep induction, they also reduce delta oscillations, suggesting that a subset of GABA_A_ receptors prevents deep restorative sleep^9,10^. Thus, identification and elimination of this confounding effect of the most widely used hypnotics, which target GABA_A_ receptors, could be beneficial in developing drugs which boost the positive effects of sleep.

Delta oscillations during NREM sleep are primarily generated by thalamocortical relay neurons^11–14^, which tend to discharge in bursts at delta frequencies when sufficiently hyperpolarized due to activation of a slow hyperpolarization-activated cation current and de-inactivation of low-threshold calcium channels^15,16^. Hyperpolarization of thalamocortical relay neurons during NREM sleep is due to the withdrawal of excitatory neuromodulatory inputs and active inhibition mediated by the major GABAergic input from the thalamic reticular nucleus (TRN)^11–13,15,16^. Recent work suggested that increased activity of TRN neurons plays a role in promoting delta power^17–19^ and NREM sleep^20–23^. However, the role of the GABAergic receptors on TRN neurons in controlling sleep oscillations is not well understood.

Here we used state-of-the-art CRISPR-Cas9 gene editing to test the hypothesis that GABA_A_ receptors on TRN neurons suppress NREM sleep delta oscillations.

## Results

### Targeting the TRN for localized alpha3 (α3) KD

**Fig. 1:**
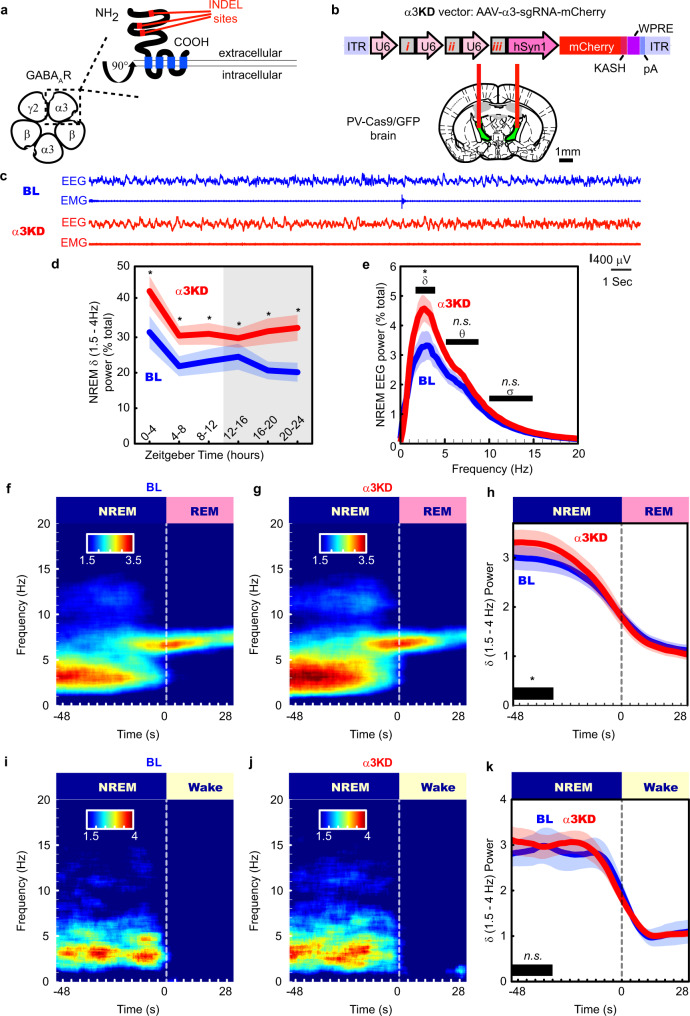
α3KD in PV + TRN neurons increased NREM 1.5–4 Hz delta power, especially at NREM to REM transitions. **a** The γ-amino butyric acid receptor type-A (GABA_A_R) is a pentameric heteromeric ion channel. CRISPR-Cas9 abscission was directed to three locations (insertion/deletion; INDEL sites) of the gene which correspond to the large extracellular region of the α3 subunit, a necessary structural as well as ligand binding component in GABA_A_Rs of the TRN. **b** Adeno-Associated Viral (AAV) vectors encoding three separate single-guide RNAs (*i, ii & iii*), each driven by its own U6 promoter, and the marker protein mCherry driven by the human synapsin (hSyn1) promoter, were injected into the TRN region of PV-Cas9/GFP mice in vivo via guide cannulas. [Adapted from Franklin and Paxinos^59^, with permission from Elsevier]. **c** Examples of frontal electroencephalogram (EEG) and electromyogram (EMG) recordings during a NREM period (ZT0-4) in baseline (BL; blue) and following α3KD (red), in a single mouse. Panels **d**–**k** present grand average data from all 6 α3KD mice. **d** Compared with their baseline (BL) recordings, α3KD mice had higher non-rapid eye movement sleep (NREM) delta (δ, 1.5–4 Hz) power. ZT0-4, *p* = 0.0176; ZT4-8, *p* = 0.0355; ZT8-12, *p* = 0.0335; ZT12-16, *p* = 0.0388; ZT16-20, *p* = 0.0275; ZT20-24, *p* = 0.0221. Significance was determined using two-tailed paired t-tests with Hommel corrected *p*-values. Thick lines indicate mean; envelopes indicate SEM. The shaded area indicates the 12 h dark period. **e** Only NREM delta (δ, 1.5–4 Hz) power is significantly different between BL and α3KD records (*p* = 0.0035, two-tailed paired *t* test), slow waves (0.5–1.5 Hz), theta (θ, 5–9 Hz) and sigma (σ, 10–15 Hz) power were not affected. The power profile from NREM in ZT-0-4 is shown here, the time period where we saw the largest effect. Significance was determined using two-tailed paired *t*-tests. Thick lines indicate mean; envelopes indicate SEM. **f** Baseline time-frequency power dynamics presents high delta power in NREM leading to a transition to rapid eye movement sleep (REM; data from the whole 12 h light period). **g** After α3KD, the high delta in NREM before a transition to REM was increased. **h** Compared with their BL levels (blue), α3KD mice (red) had higher delta power in the NREM before a transition to REM [t *(5)* = 2.14, *p* = 0.04]. Significance was tested using a one-tailed paired *t*-test. Thick lines indicate mean; envelopes indicate SEM. **i** BL time-frequency power dynamics presents high delta power in NREM leading to a transition to wake as well. **j** α3KD did not increase this delta power that occurs during NREM before a transition to wake. **k** Compared with BL (blue), α3KD (red) did not lead to a change in delta power in the NREM before a transition to wake [t *(5)* = 0.23, *p* = 0.41]. Significance was tested using a one-tailed paired *t*-test. Thick lines indicate mean; envelopes indicate SEM. *p < 0.05, *n.s*. indicates not significant. Color scales represent normalized power (power at time/power from wakefulness).

In the adult brain, most GABA_A_Rs consist of two α subunits (α1–6), two β subunits (β1–3), and one γ subunit (γ1–3)^24^ (Fig. 1a). The α subunit is a necessary component of the GABA_A_R, required for assembling a functional receptor^25^. In the mouse thalamus, all synaptic GABA_A_Rs in thalamocortical relay nuclei contain the α1 subunit, whereas GABA_A_Rs in the TRN contain the α3 subunit^26^. To introduce a brain region and cell-type-specific ablation of the α3 subunit gene, we first generated mice which expressed the Cas9 endonuclease in the major subset of TRN neurons which contain the calcium-binding protein parvalbumin (PV) by crossing PV-Cre mice with Rosa26-Lox-stop-lox-Cas9-GFP mice to produce PV-Cas9-GFP offspring. Next, we analyzed the gene sequence of the α3 subunit and selected three loci close to the start codon as target regions expected to maximize CRISPR-Cas9 mediated ablation (Fig. 1a). Our target regions were within the extracellular domain (Fig. 1a), the major ligand binding component, forming parts of the GABA binding site and the benzodiazepine binding site^27^. We then constructed an adeno-associated viral (AAV) vector to target the α3 subunit (AAV5-α3-sgRNA-mCherry) by introducing the sequences for the single-guide RNAs (sgRNAs) into an AAV vector plasmid, each driven by the U6 promoter paired with mCherry as a red fluorescent marker (Fig. 1b). To test the effect of α3 knockdown (α3KD) on sleep and spectral activity, we recorded cortical oscillations using frontal electroencephalographic (EEG) electrodes and nuchal muscle electromyographic (EMG) electrodes before and after we introduced AAV-α3-sgRNA-mCherry into the TRN via chronically implanted guide cannulae (Fig. 1b).

### NREM delta wave power is enhanced by α3KD in the TRN

α3KD in TRN PV neurons resulted in a marked increase in NREM slow wave activity (0.5–4 Hz, which was most pronounced at the beginning of the mouse sleep period (ZT0-4, 47.5 ± 21.8% change, *t* (5) = −5.159, *p* = 0.0036). The increase in slow wave activity was similar in magnitude to the increase observed in the first four hours following six hours of sleep deprivation^28^, even though the mice here were not sleep deprived.

**Fig. 2:**
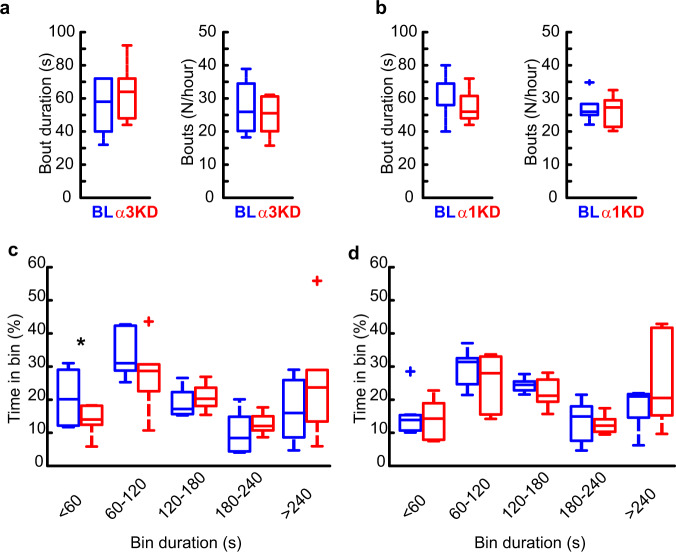
α3KD, but not α1KD, in PV + TRN neurons decreased time spent in the shortest (<60 s) NREM bouts. **a** Compared with their baseline conditions (BL; blue), α3KD (red) mice (*n* = 6) did not have altered durations or number of NREM bouts. **b** α1KD mice (*n* = 7) also did not have altered durations or number of NREM bouts. **c** The proportion of time spent in short bouts lasting <60 s was significantly reduced in α3KD mice (two-tailed paired *t*-test, *p = 0.03), but no other bins showed any differences between BL and α3KD (*n* = 6). **d** The proportion of time spend in bouts was unchanged in α1KD mice (*n* = 7). Box plots: center lines represent median values, 75^th^ percentiles are box tops and 25^th^ percentiles are box bottoms. + symbols represent outliers defined as >1.5x the interquartile range away from box tops or bottoms. Data from whole 12 h light period.

The 0.5–4 Hz EEG band includes both cortically-generated slow waves (typically 0.5–1 Hz or 0.5–1.5 Hz)^29–32^ and delta oscillations (1.5–4 Hz), which are largely thalamically generated^29–32^. More fine-grained analysis of our data to separate out these two components revealed that significant increases were seen in the 1.5–4 Hz delta range (Fig. 1d, e and Supplementary Fig. 1) as well as in narrower bands including the delta2 (2.5–3.5 Hz) band which is sensitive to sleep deprivation^31^ (Supplementary Table 1) whereas there was no significant change in slow oscillation (0.5–1.5 Hz, 0–1 Hz or 0.5–1.75 Hz) EEG bands (Supplementary Table 1). 6/6 mice with > 85% transduction of TRN PV neurons showed the increase in delta power (Supplementary Fig. 1). We found no significant change in any other frequency bands in NREM (Fig. 1e), and no changes in any frequency bands during wakefulness or REM sleep (Supplementary Fig. 2). Only modest changes in the amount of NREM sleep itself were observed, with more NREM only in the first four hours of the dark period when mice are mostly awake. (BL 23.2 ± 2.4% vs KD 32.4 ± 2.8%, *t* (5) = −4.7472, *p* = 0.005). Moreover, we found no changes in the duration or frequency of NREM bouts (Fig. 2a). However, we observed a significant reduction in the proportion of shortest bout durations (Fig. 2c), suggesting more consolidated NREM sleep following α3KD. REM sleep was unchanged [duration: BL = 35(1) vs α3KD = 38(3.79); bouts/hour: BL = 6.59(0.41) vs α3KD = 6.42(0.59)]. Analysis of sleep spindles using a recently validated algorithm^33^ did not identify any difference in spindle density, frequency or duration (Supplementary Fig. 3). No NREM delta effects were observed in four negative control mice three of which showed no AAV-α3-sgRNA-mCherry transduction in TRN and one with only 66% transduction of TRN PV neurons (Supplementary Fig. 4).

In humans, delta oscillations are most prominent in the deepest stage of NREM sleep, N3. However, in mice NREM is not generally split into stages. Nevertheless, mouse NREM probably also has degrees of depth which are not evident using standard scoring approaches. In humans, arousal threshold increases with depth of NREM sleep; humans are more likely to awaken from the lighter stages N1 or N2^34^. Thus, we analyzed delta oscillations prior to NREM → REM and NREM → wakefulness transitions. The heightened delta power associated with α3KD was only evident during NREM sleep preceding transitions to REM sleep (Fig. 1f–h) [means (standard error (SEM): BL = 2.98 (0.24), α3KD = 3.3 (0.26); 11.5% change for all transitions occurring during the light period (±5.56); t *(5)* = 2.14, *p* = 0.04]. No difference was apparent in NREM sleep before transitions to wake (Fig. 1i–k). Similarly, no change in delta power was seen during NREM sleep in the initial phase of transitions from wakefulness to NREM sleep (Supplementary Fig. 5). We also found that increased delta power prior to NREM → REM transitions was not apparent in the negative control group with either no or poor TRN targeting (Supplementary Fig. 4).

### Enhanced NREM delta corresponds to longer NREM bouts

**Fig. 3:**
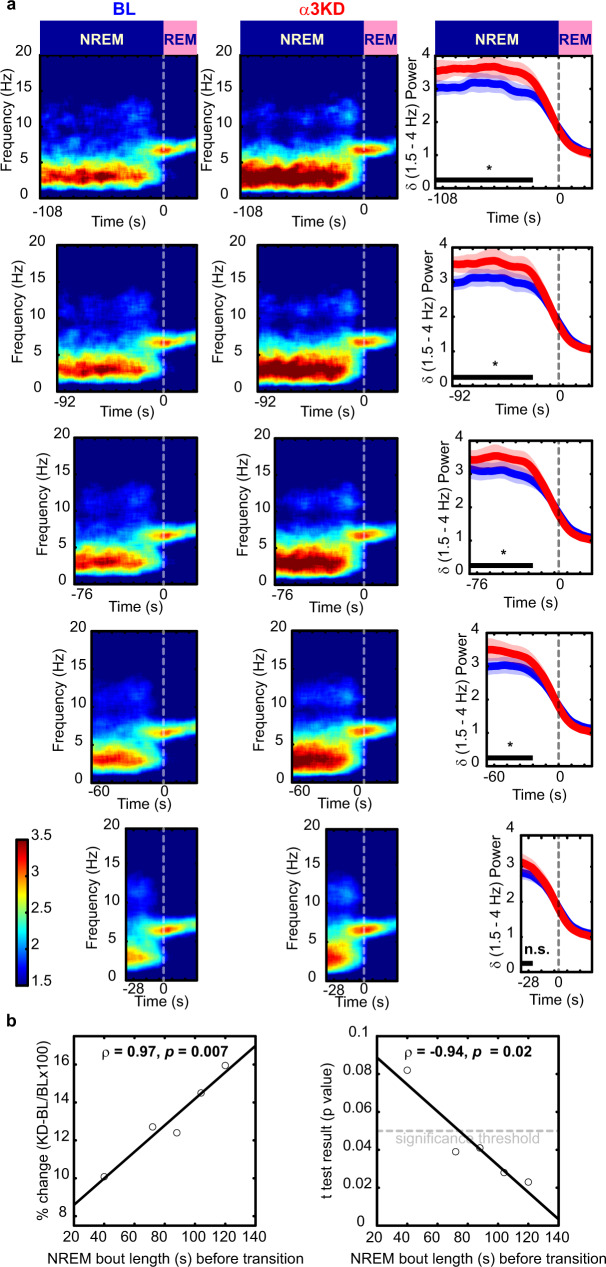
α3KD caused the largest delta (δ, 1.5–4 Hz) effect in the longest NREM bouts before REM. **a** Analysis of non-rapid eye movement sleep (NREM) to rapid eye movement sleep (REM) transitions reveals larger delta power increases in longer NREM episodes, baseline (BL; blue) vs α3KD (red). Periods with stable delta (black bars), were tested for significance (one-tailed paired *t*-tests, *p < 0.05, n.s. not significant; *p*-values: 0.023; 0.028; 0.041; 0.039; 0.082.). The 24 s prior to NREM-REM transitions, when delta decays, were not included in statistical analyses. **b** Percent change in NREM delta power was positively correlated with NREM bout length prior to REM. *P* values from *t*-tests (BL vs α3KD) were negatively correlated with NREM bout length prior to REM as determined using Pearson’s linear correlation, two-tailed, ρ is Pearson’s linear correlation coefficient. Data from 12 h light period. *N* = 6. Color scales represent normalized power (power at time/power from wakefulness).

Sleep is more fragmented in mice than in humans. Thus, we reasoned that long NREM bouts might be required to observe large increases in delta. Further analysis of NREM-REM transitions revealed larger increases in delta power within sustained NREM periods prior to transitions to REM (Fig. 3a). Interestingly, heightened delta was seen at the onset and throughout the NREM bout, rather than gradually increasing as a NREM bout persists (Fig. 3a). Delta power levels were higher in longer NREM bouts and were increased more by the α3KD. In fact, there was a highly significant linear relationship whereby the longer the NREM duration preceding a NREM-REM transition, the larger the increase in delta power, as measured by percent changes (Fig. 3b). These data also suggested the possibility that the difference in the delta increase observed between NREM-REM and NREM-wake transitions might be explained, at least in part, by a shorter NREM episode duration prior to NREM-wake transitions compared with the longer NREM preceding REM sleep. This prediction was confirmed since NREM episodes leading to wakefulness were shorter (76.3 ± 7.1 s) than those which transitioned into REM sleep (253.2 ± 30.9 s).

### Widespread α3KD in TRN determines elevated NREM delta power

**Fig. 4:**
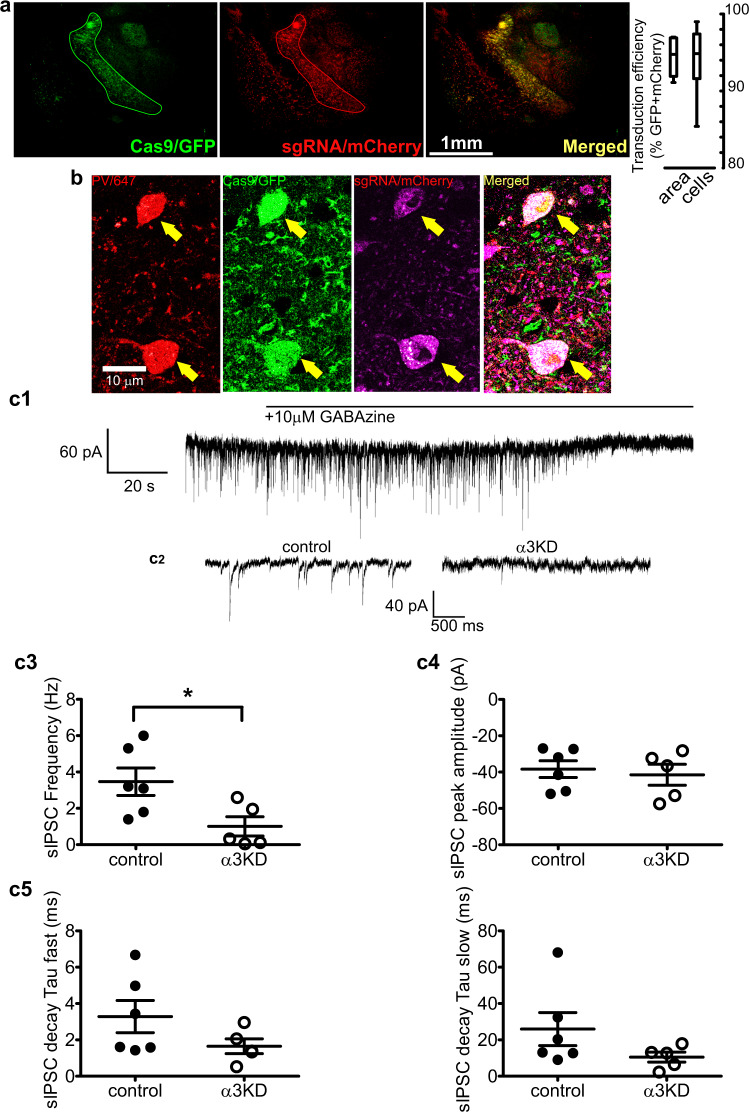
α3KD in PV+ TRN neurons was validated by histology and in vitro electrophysiology. **a** GFP indicates rich Cas9 expression within the TRN region (green outline), TRN projections to thalamocortical nuclei and sparce distal expression in the globus pallidus (GP). mCherry reveals widespread transduction of the TRN region by the AAV vector delivering sgRNAs (red) with many of the cells in the area co-expressing both markers (merged; yellow). Percentages of target cells and target area that co-express markers reveal widespread delivery of sgRNAs to target TRN PV neurons in the mice used for in vivo studies. Further quantification of transduction is given in Supplementary Tables 2 and 3. Box plots: center lines represent median values, 75^th^ percentiles are box tops and 25^th^ percentiles are box bottoms, bars represent maximum and minimum. (*n* = 6). **b** High magnification (60x) confocal images show triple co-localization of PV (immunohistochemical stain; red), Cas9 (GFP; green), and sgRNA (viral transduction indicated by mCherry; magenta), demonstrating successful targeting of PV+ neurons within the TRN. Micrographs are representative of *n* = 2. **c**1 Inward-going spontaneous inhibitory postsynaptic currents (sIPSCs) recorded from TRN PV neurons using a high chloride intracellular solution in the presence of ionotropic glutamate receptor antagonists are blocked by the selective GABA_A_ receptor antagonist, GABAzine (10 µM). **c**2 Compared with PV+ TRN neurons without KD (control), sIPSCs in α3KD PV+ TRN neurons were reduced (α3KD). **c**3 Compared with control recordings from PV+ TRN neurons without KD (left), the frequency of sIPSCs in α3KD PV+ TRN neurons was significantly reduced (right). *p = 0.031, unpaired two-tailed *t*-test. **c**4, 5 Compared with control recordings from PV+ TRN neurons without KD, residual sIPSCs in α3KD PV+ TRN neurons (right) had unaltered amplitude (**c**4) or decay time constants (**c**5). **c**3–**c**5 broad lines indicate means, error bars represent SEM control: *n* = 6; α3KD *n* = 5.

Selective deletion of α3 subunits in TRN PV neurons requires the combination of selective expression of Cas9 in PV neurons and sgRNA targeting α3 subunits in the same cells. mCherry (red; marker of sgRNA) was expressed in the majority of TRN PV neurons (green) within the core of the injection site (Fig. 4a). In the six α3KD-confirmed mice, we found a high percentage of PV + TRN neurons (GFP+) were transduced by AAV-α3-sgRNA-mCherry (mCherry+: 93.9 ± 2.0%), and a large proportion of the TRN (94.4 ± 1.0%) area was covered (Fig. 4a; Supplementary Table 2). Analysis of off-target viral spread assessed by mCherry+ cells which were also GFP+ revealed only 5% medially and 3.9% laterally. All cases of off-target mCherry/GFP colocalization were found exclusively in the anterior dorsal thalamic nucleus and globus pallidus (Supplementary Table 3). Neither of these regions express substantial amounts of α3 subunit containing GABA_A_ receptors^35–39^. Thus, our functional effects are highly likely to be due to effects on the TRN. In preliminary work prior to in vivo experiments, we confirmed that Cas9 expression (marked by GFP co-expression) was selective for PV neurons by immunohistochemical staining for PV (Fig. 4b), consistent with the previously published validation of Cas9 selective expression in PV+ neurons in this mouse model^40^.

### α3KD causes a functional ablation of sIPSCs in TRN neurons

In a separate group of mice, we verified a functional ablation of GABA_A_ receptors in whole-cell patch-clamp recordings from TRN PV neurons in vitro. Recordings were performed from adult mice (>2.5 months). In contrast to earlier in development^41^, at this age in mice there is little evidence for functional intra-TRN chemical synapses^41,42^. Thus, the GABA_A_ receptor mediated events we record in TRN PV neurons likely arise from extra-TRN inputs arising in the basal forebrain, lateral hypothalamus and globus pallidus^20,21,43^. In control voltage-clamp recordings from TRN PV neurons held at −70 mV in PV-tdTomato mice (which serve as wild type controls with a visual marker of the correct cell phenotype), spontaneous inhibitory postsynaptic currents (sIPSCs) were observed in the presence of glutamate receptor antagonists (20 µM 6-cyano-7-nitroquinoxaline-2,3-dione +50 µM D-(2R)-amino-5- phosphonopentanoic acid) and were abolished by a GABA_A_ receptor antagonist, GABAzine (10 µM) (Fig. 4c1). To enhance the driving force for chloride, recordings were made using a patch solution with a high chloride concentration. Thus, IPSCs were detected as inward currents (Fig. 4c1, 4c2). In PV-Cas9 mice, the frequency of sIPSCs were significantly reduced in recordings from green (PV-Cas9/GFP) and red (transduced with AAV-α3-sgRNA-mCherry) fluorescent TRN neurons one month post-injection, whereas the amplitude was unaltered [Frequency: PV-tdTomato: 3.47 ± 0.75 Hz (*N* = 6 from four animals); PV-Cas9+AAV-α3-sgRNA: 1.01 ± 0.53 (*N* = 5 from four animals); *t*(9) = 2.560, *p* = 0.031, *t*-test, (Fig. 4c3). Amplitude: PV-tdTomato: −38.4 ± 4.6 pA (*N* = 6); PV-Cas9+AAV- α3-sgRNA: −41.5 ± 5.8 pA (*N* = 5), *t*(7) = 0.4426, *p* = 0.6793, *t*-test; (Fig. 4c4). The bi-exponential decay of residual sIPSCs in PV + TRN neurons in PV-Cas9 mice with α3KD was not significantly different from that in control PV-tdTomato mice [Fig. 4c5; Fast decay time constant: PV-tdTomato 3.29 ± 0.89 ms; PV-Cas9+AAV- α3-sgRNA: 1.64 ± 0.41 (*N* = 5), *p* = 0.1524, *t*-test. Slow decay time constant: PV-tdTomato 26.0 ± 9.1 ms; PV-Cas9+AAV- α3-sgRNA: 10.6 ± 2.7 (*N* = 5), *p* = 0.1692, *t*-test]. Furthermore, residual sIPSCs retained sensitivity to an α3 selective positive allosteric modulator, TP003 [1 µM TP003, slow component of bi-exponential decay time increased by 19.2 ± 6.5%, *p* = 0.071, *N* = 3, paired *t*-test], suggesting that other α subunits were not upregulated in response to the α3KD. No change was observed in the holding current (PV-tdTomato control: −141.0 ± 26.0 pA; PV-Cas9+AAV-α3-sgRNA −84.4 ± 7.6 pA, *p* = 0.1155) indicating that knockdown of α3 subunits did not alter the passive membrane properties of TRN PV neurons.

### No effect by control α1KD

**Fig. 5:**
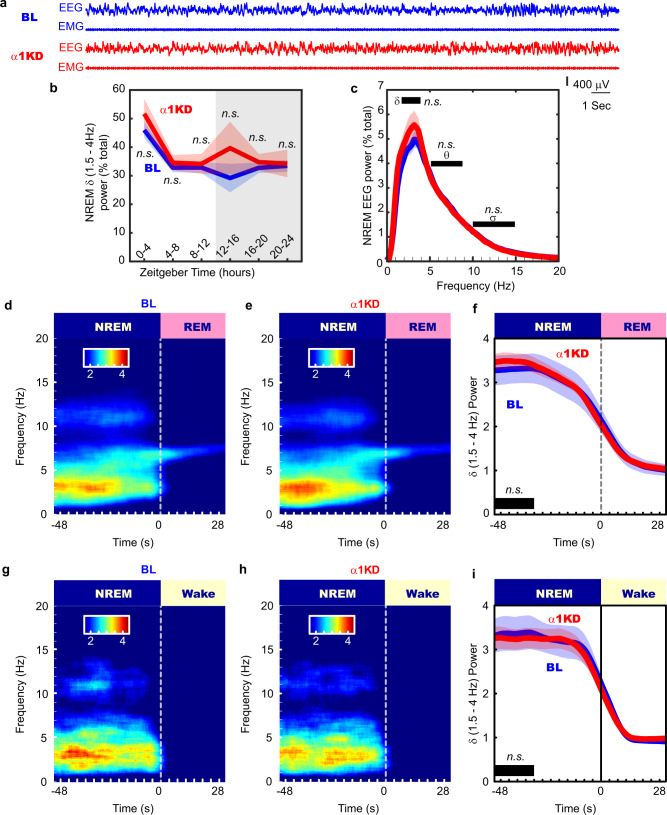
The control cohort with α1KD in PV + TRN neurons displayed no changes to NREM or wake time, or delta (δ, 1.5–4 Hz) power in any states, including transitions from NREM to REM, in the light (inactive) period. **a** Examples of frontal electroencephalogram (EEG) and electromyogram (EMG) recordings during a NREM period (ZT0-4) in baseline (BL; blue) and following α1KD (red), from a single mouse. **b** Compared with their baseline (BL) recordings, α1KD mice had unchanged non-rapid eye movement sleep (NREM delta power ZT0-4, *p* = 0.2; ZT4-8, *p* = 0.32; ZT8-12, *p* = 0.58; ZT12-16, *p* = 0.42; ZT16-20, *p* = 0.47; ZT20-24, *p* = 0.88. Significance was determined using two-tailed paired *t*-tests. Thick lines indicate mean; envelopes indicate SEM. The shaded area indicates the 12 h dark period. **c** NREM delta (δ) theta (θ) and sigma (σ) power were not different between BL and α1KD records, shown here is the power profile from NREM in ZT-0-4 when we saw the largest effect in the α3KD mice. Significance was determined using two-tailed paired *t*-tests. Thick lines indicate mean; envelopes indicate SEM. **d** Baseline time-frequency power dynamics reveal high delta power in NREM leading to a transition to rapid eye movement (REM) sleep. **e** After α1KD, the high delta power in NREM before a transition to REM is the same as in baseline records. **f** Compared with baseline (blue), α1KD (red) mice had unaltered delta power in the NREM before a transition to REM [t *(6)* = 0.67, *p* = 0.26]. Significance was tested using a one-tailed paired *t*-test. Thick lines indicate mean; envelopes indicate SEM. **g** Baseline time-frequency power dynamics reveals high delta power in NREM leading to a transition to wake. **h** α1KD did not increase the delta in NREM before a transition to wake. **i** Compared with baseline (blue), α1KD (red) mice had unchanged delta power in the NREM before a transition to wake [t *(6)* = −0.36, *p* = 0.63]. Significance was tested using a one-tailed paired *t*-test. Thick lines indicate mean; envelopes indicate SEM. *n.s*. indicates not significant. **d**–**i** show data from whole 12 period of light. **b**–**i** *N* = 7. Color scales represent normalized power (power at time/power from wakefulness).

In another group of mice, we performed the same in vivo experimental protocol with a control AAV vector targeting the GABA_A_R α1 subunit. The TRN is devoid of α1 subunits, so this experiment controls for non-specific genetic cutting. In these mice (*n* = 7), we found no change in the amount of NREM delta power (Fig. 5a, b), or other bands in NREM (Fig. 5c) following the α1KD. No frequency bands of wakefulness or REM sleep were altered either. The time-frequency analysis at NREM-REM transitions (Fig. 5d–f) and at NREM-Wake transitions (Fig. 5g–i) showed no changes. There were also no changes to the duration or frequency of NREM bouts (Fig. 2b), or the proportions of bout durations (Fig. 2d). Our histologic protocol confirmed a similar degree of targeting success (Supplementary Fig. 6) as in the α3KD.

## Discussion

In this study, we used cell-type and region-specific CRISPR-Cas9 gene editing in vivo to test the functional role of GABAergic inhibition onto TRN neurons in controlling sleep physiology. We found that knockdown of α3-containing GABA receptors, confirmed using in vitro recording of sIPSCs, selectively enhances the power of NREM delta oscillations during the sleep period of mice. Further analyses identified long NREM episodes prior to NREM-REM transitions as being particularly strongly affected by α3KD. The selectivity of our manipulations was confirmed by control experiments with absent or low transduction and experiments targeting a closely related subunit, α1, which is not expressed by TRN neurons.

There is an emerging consensus that depolarization of TRN neurons during NREM is an effective way to promote deep sleep^17–23^. TRN neurons receive GABAergic inputs from basal forebrain, lateral hypothalamus and globus pallidus, and several of these neuronal groups maintain a high discharge rate during NREM sleep^20,21,43^. Previous in vitro work^44^ showed that disinhibition of TRN neurons via local pharmacological blockade of GABA_A_ receptors in TRN enhances GABAergic inhibition in thalamocortical neurons. Thus, withdrawal of these inputs by removing their postsynaptic targets will lead to a higher discharge rate of TRN neurons during NREM sleep, particularly during deeper stages of NREM prior to REM sleep when excitatory inputs from brainstem aminergic cell groups wane. In turn, increased activity of TRN GABAergic neurons will lead to hyperpolarization of thalamocortical relay neurons, bringing their membrane potential into the correct range to generate delta oscillations. This interpretation of our results is consistent with previous work which suggested that modulating the polarization level and discharge rate of TRN neurons affects delta oscillations and NREM sleep^17–22^. In particular, tonic optogenetic excitation of TRN neurons increased burst discharge of thalamocortical relay neurons at delta frequencies and increased cortical delta power^17^. Taken together, our in vitro electrophysiology experiments and the prior literature suggest that the increase in cortical delta power in our experiments is likely explained by increased tonic discharge of TRN PV neurons due to reduced extra-TRN inhibitory influences and enhanced delta-frequency bursting of thalamocortical neurons. We also note that reduced burst discharge of TRN neurons due to knockout of Cav3.3 channels or chemogenetic inhibition of TRN neurons can lead to increased delta power^23^ but in contrast to the results reported here and in previous studies^17–19^, these manipulations reduce the power of slow oscillations and sleep spindles^23^. Given the functional importance of slow oscillations and sleep spindles, manipulations which increase TRN activity during sleep may be more beneficial than those which reduce burst discharge or cause strong hyperpolarization.

Our findings differ from previous work which examined constitutive global α3 subunit knockout (KO) mice^25,45^. In the constitutive KO there was no reduction in sIPSC frequency compared to controls in TRN neurons; in fact, there was a modest increase in frequency, plus a significant increase in amplitude and alterations in kinetics and pharmacology, suggesting developmental compensation. Conversely, we show a significant reduction in sIPSC frequency. Here, the ablation was performed in the adult brain, so developmental compensation was circumvented, which is evident by the lack of change in the amplitude of residual IPSCs or their decay time constant, as well as the fact that residual sIPSCs retained sensitivity to an α3 selective positive allosteric modulator. Therefore, functional ablation of α3 subunits in adults was feasible with the CRISPR-Cas9 approach and, importantly, allowed us to unravel the role of the α3 subunits in sleep-wake patterns and EEG profiles. Despite marked changes in GABAergic transmission in the TRN in the constitutive global α3 knockout, there was no change in the intrinsic membrane properties of TRN neurons or in the properties of glutamatergic inputs from thalamocortical relay neurons^25,45^. Similarly, we found no change in the holding current of TRN PV neurons in vitro. Thus, alterations in GABAergic transmission in TRN do not appear to lead to compensatory alterations in the thalamocortical circuitry which generates delta oscillations.

In conclusion, CRISPR-Cas9 cell and region-specific gene editing of α3 subunits in adult mice identified a functional role of GABA_A_ receptors on TRN PV neurons in regulating deep NREM sleep. Pharmacological agents which allosterically increase the activity of GABA_A_ receptors containing the α1 or α3 subunits are widely used hypnotics. Unfortunately, they promote light sleep with reduced delta power^9,10,46^. Our results suggest that the delta suppressing effect of z-drugs may come from potentiating the α3 containing GABA_A_Rs of the TRN (i.e., the opposite effect that we report here; α3KD leads to increased delta power), a sleep-regulating region which has one of the highest densities of α3 subunits in the brain. Clinically, this is a problem because high NREM delta waves of deep sleep are restorative, important for memory consolidation^47^ and clearance of toxic metabolites^3^. Here knockdown of α3 subunits on TRN neurons enhanced deep sleep while not negatively affecting other sleep oscillations or wake power spectra. Mainstay sleep medicines which potentiate GABA_A_ receptors, termed z-drugs, suppress delta waves. Each drug preferentially targets various isoforms. For example, Zolpidem and eszopiclone both bind to α1 α2 and α3, eszopiclone additionally binding α5^48^. Interestingly GF-015535-00, not currently approved for clinical use, is highly selective for α1 and causes far less delta suppression than currently approved z-drugs^49^. An ideal next-generation hypnotic agent may sedate by positive allosteric modulation of α1 subunits (as with conventional z-drugs); but simultaneously boost delta waves by negatively modulating α3 subunits which are highly expressed in TRN of humans/primates^50,51^, as well as mice. This mechanism could enhance the properties of natural restorative delta oscillations of NREM sleep in manner that current hypnotics do not.

## Methods

### Mice

To target our Clustered Regularly Interspersed Short Palindromic Repeats Knock Down (CRISPR KD) selectively to the major subset of Thalamic Reticular Nucleus (TRN) neurons which express Parvalbumin (PV) we crossed male Rosa26-lox-stop-lox-Cas9/GFP (Jackson Labs stock # 026175) mice with female PV-Cre (Jackson Labs stock # 017320) mice, generating mice with the key CRISPR enzyme Cas9 and a green fluorescent protein (GFP) reporter expressed selectively in PV neurons, PV-Cas9/GFP mice. For one control group used for in vitro sIPSCs recordings, we used PV-tdTomato mice generated by crossing male Rosa26-lox-stop-lox-tdTomato (Jackson Labs stock # 007914) mice with female PV-Cre mice. 3–8 month-old mice of both sexes were used for in vivo and in vitro experiments. No obvious sex differences were observed so data were pooled. Mice were housed with a 12 h:12 h light:dark cycle with lights on at 7am, at ambient temperatures 26–34 °C and 30–70% humidity. Food and water were available ad libitum. All experiments were approved by the Institutional Animal Care and Use Committee of VA Boston Healthcare System and conformed to National Institute of Health, Veterans Administration and Harvard Medical School guidelines. The work was carried out under protocols 236-B-042916, 359-B-041618 & 400-W-110419.

### Adeno-associated viral (AAV) vectors

For this study, the PX552 plasmid described and validated by Swiech et al.^52^ (Addgene plasmid # 60958; http://n2t.net/addgene:60958; RRID:Addgene_60958) was modified to encode triple U6-sgRNA cassettes each targeting a distinct locus of the target genes. The GFP sequence was replaced by the sequence encoding the red fluorescent protein, mCherry, since GFP was already expressed in PV neurons in the PV-Cas9/GFP mice used for experiments. This custom plasmid became our backbone for subsequent design of our control vector.

#### AAV-α3-sgRNA-mCherry

To selectively inactivate the gene encoding the only α subunit (α3) of GABA_A_ receptors expressed in TRN^26^, we used the above custom-designed AAV vector to deliver three sgRNAs targeting the gene encoding the α3 GABA_A_ subunit *(Gabra3)* within the TRN: (*Gabra3;* NCBI Reference Sequence: NM_008067.4): *i:* 5′ TCTTCACTAGAATCTTGGAT 3′ *ii:* 5′ GGACCCTCCTCTATACAATG 3′ and *iii* 5′ TTGTTGGGACAGAGATAATC 3′. We found in silico that the α3-sgRNAs had no off-targets in the mouse genome^53^. The CRISPR design tools http://crispr.mit.edu/ and http://chopchop.cbu.uib.no/^54^ were used to aid initial identification of possible sgRNA sequences corresponding to the N-terminal domain (Fig. 1a). The sgRNA candidate sequences were manually selected by alignment analysis (Bioedit; open-source software, Ibis Therapeutics, CA) to confirm that they did not target the closely related gene encoding the α1 subunit; (*Gabra1;* NCBI Reference Sequence: NM_010250.5). Though not found in TRN, α1 is present in the thalamus^26^ and we wanted to avoid off target GABA_A_ receptor KD, which we viewed as the only tangible confound beyond non-specific CRISPR-Cas9 abscission. All three α3-sgRNA showed minimal homology with α1 and was not indicated to be an off-target site in silico.

#### AAV-α1-sgRNA-mCherry

We designed another vector bearing three sgRNAs targeting the α1 gene of GABA_A_ receptor. One sgRNA with low homology to α3 but perfect homology to α1 with a PAM sequence and the other two mock-sgRNAs with perfect homology to non-PAM bearing areas of α1 gene were chosen. *i:* 5′ CTCATTCTGAGCACACTGTC 3′ *ii*, 5′ TTTTTCCGTCAAAGTTGGAA 3′ *iii* 5′ TTGGACAAACAGTTGACTCT 3′. All three sequences from mouse α1 gene showed no off-targets in silico in the mouse genome^53^. We used snapgene software for plasmid design, construction was outsourced to GenScript (New Jersey, United States; www.genscript.com/) and validated by restriction mapping by gel electrophoresis and sequencing. Plasmids, thus validated, were packaged into AAV5 by the University of North Carolina vector core facility. Vector titers (~1–4 × 10^12^ particles/ml) were determined by dot-blot analysis and used for microinjections into the TRN (Fig. 1b).

### Stereotaxic surgery and AAV microinjections into TRN

Executing the selective CRISPR KD of α3 subunits in TRN PV neurons requires expression of the sgRNAs targeting α3 subunits combined with the Cas9 expressed in PV neurons of the transgenic mice. We achieved this combination by stereotaxic injection of the AAVs expressing sgRNAs into the TRN of the PV-Cas9/GFP mice. Using a Kopf stereotaxic frame, we chronically implanted cannulas bilaterally above the anterior TRN (Plastics One; Connecticut, United States), Part # C315G/SPC, total length 12 mm), with retainers inserted (Plastics One, Part # C15I/SPC), at AP −0.7 mm, ML ±1.4, DV −1.5. The coordinates for cannula location were selected to be 2 mm above the TRN to allow subsequent microinjection into TRN without extensive damage to TRN. We targeted anterior TRN since anterior TRN neurons project to thalamocortical regions which innervate anterior cortex where our EEG recording electrode was located^55^. To record cortical electrical activity, bilateral frontal neocortical EEG screw electrodes (Pinnacle Technology Inc.; Kansas, United States; Part # 8403) were placed at AP +1.9 mm, ML ±1.5 with a reference electrode at AP −3 mm, ML +2.7 and a ground electrode at AP −6 mm ML = 0 respectively and soldered to a headmount (Pinnacle Technolology Inc.; Part # 8201-SS). EMG electrodes were placed in the nuchal muscle. All the chronically implanted components were secured with dental cement (Keystone industries, Bosworth Fastray; Part # 0921378).

Following one full week of recovery from cannula/electrode implantation, and collection of baseline EEG/EMG records (see next section), AAV microinjections were made into the TRN. Mice were anesthetized by isoflurane (1.5–4% in O2) and depth of anesthesia was monitored by breathing rate, pedal withdrawal and tail pinch reflexes. A 5 μl Hamilton syringe (Part # 87908, Model 75 SN SYR with 33 g cemented needle) loaded with viral solutions was lowered through the cannula, 2 mm beyond the cannula tip, into the TRN (DV −3.5). 1 μl microinjections were delivered at 0.05 μl/minute using a micropump (KD Scientific Legato 130, Massachusetts, United States). Doses of Meloxicam (5 mg/kg; intraperitoneal) were given immediately after surgery and again 22–24 h later, to mitigate any pain associated with the surgery. One month following AAV injection, EEG/EMG signals were again recorded and compared to baseline recordings.

### Electroencephalogram (EEG)/Electromyogram (EMG) recordings

To study sleep wake-states and thalamocortical oscillations, we recorded EEG and EMG using Pinnacle Technology Inc. 3 channel (2 EEG/1EMG) systems for mice (Part # 8200-K1-SL), using its acquisition software (Sirenia Acquisition). Mice were tethered to the system via mouse pre-amplifiers (Pinnacle Technology Inc. Part # 8202-SL) and 24-hour recordings were collected between zeitgeber time 0–24 following a 48-hour period of habituation to the recording apparatus. EEG/EMG data was sampled at 2 kHz, amplified 100x and low pass filtered at 600 Hz. In one mouse from the α3KD cohort and one mouse from the control cohort which lacked transduction of PV + TRN neurons, we missed data from ZT0-ZT2. In one mouse from the control cohort which lacked transduction of PV + TRN neurons we missed data from ZT0-ZT3. These were due to software malfunctions. We dealt with this by continuing the recordings into the subsequent day and using the same ZT hours to replace the missing data.

### Sleep-wake scoring and EEG analysis

We manually scored sleep-wake states from EEG and EMG records using four second epochs as follows: Wake was scored when EEG showed a desynchronized low amplitude signal with muscle tone evident by a large EMG signal; the large EMG signal did not need to be phasic in appearance to be characterized as wake. NREM sleep was scored when the EEG signal showed large amplitude, slow synchronized waves, and a low EMG signal, except for very brief bursts which were considered twitching. REM sleep was scored when the EEG signal presented a repetitive stereotyped ‘sawtooth’ signal in the theta range (5–9 Hz), with a nearly flat EMG signal. Artifacts were dealt with as follows: periods that appeared to be wakefulness, but the EEG was contaminated by crosstalk with EMG signals, were scored as ‘wake-exclude’. Periods of NREM or REM with large amplitude DC shifts were extremely rare and labeled as ‘NREM-exclude’ or ‘REM-exclude’. We used all epochs, even those marked ‘exclude’, in behavioral analyses such as time in state or bout analysis, but we only included artifact free data for analyses of EEG signals, such as power spectral density or time-frequency analyses. Scoring was performed in Sirenia Sleep, and EEG signals and scored epochs were exported for further analysis in MATLAB.

Power spectral density of wake, NREM and REM was computed using the MATLAB pwelch function using an 4-second Hanning window with 50% overlap. To normalize EEG power, power from each state was normalized to the total power from the entire 24 h record. We used this approach having read methods in previous sleep-wake power analysis in mice^56^. Briefly, delta power dynamics across the 24 h record were binned in 4-hour intervals (still normalized to the full 24 h record) to ensure there were no periods devoid of NREM, all NREM bouts were used to compute NREM delta power. To produce time-frequency spectrograms for state-transition analysis, we first down-sampled the data to 40 Hz and screened for outliers, replacing values more than ten standard deviations away from the mean with zeros. We then used the multi-taper method^57^ (Chronux Toolbox; Chronux.org) function (5 tapers with 10 s sliding window in 100 ms steps). Spectra were computed for each state-transition per mouse and mean averaged. Each within-animal mean averaged time-frequency spectrum was then normalized to its average power from wakefulness (I.E. Frequency bin from spectra/Frequency bin from wake). These normalized spectra were then mean averaged across all the animals in the group for a grand mean averaged spectrogram. Mean averaged power in the delta band (1.5–4 Hz) was plotted with standard error envelopes, smoothed using a 5 s moving average window. We analyzed sleep spindles using our validated automated spindle detection algorithm^33^ and plotted them at NREM-REM transitions as mean values of corresponding 12 s periods across all transitions. For bout analysis, bout durations were binned into discrete intervals, summed, and presented as a percentage of total time in the state. We used this approach having read methods in previous sleep-wake bout analysis in mice^58^.

### Histology

Mice were transcardially perfused with 10 ml phosphate buffered saline (PBS) followed by 10 ml of 10% formalin for fixation. Brains were extracted and subsequently immersion-fixed in 10% formalin for 1–2 days, followed by 30% sucrose solution in PBS before tissue was sliced at a thickness of 40 µm on a freezing microtome (Leica Biosystems, Illinois, United States).

We first confirmed previous findings^40^ that Cas9 was expressed selectively in TRN PV neurons, by performing immunohistochemistry for PV in coronal sections containing TRN from PV-Cas9/GFP mice. Free-floating sections in wells were treated with PV primary antibodies sheep anti-PV (1:150; AF-5058; R & D Systems, Minneapolis, MN) and far-red secondary antibodies (donkey anti-sheep IgG conjugated to AlexaFluor 647; 1:250; ab 150179; Abcam, Inc. Waltham, MA). GFP signal was enhanced using mouse anti-GFP antibody (1:300; MAB3580; EMD Millipore, Burlington MA) and the green secondary antibody donkey anti-mouse IgG conjugated to AlexaFlour 488 (1:500; #A-21202; ThermoFisher Scientific, Waltham, MA). Confocal imaging was performed for triple fluorescence (mCherry, AF488, AF647) using 60x oil objective of Leica Dmi8 confocal microscope and LAS X software.

For histological verification of transduction efficiency, sections were mounted on microscope slides and coverslipped using Vectashield Hard Set mounting medium (Part # H-1400, Vector Labor). Images were collected on a Zeiss Image2 microscope, outfitted with a Hamamatsu Orca R2 camera (C10600) and Stereo Investigator software (MBF Bioscience). For our in vivo sleep-wake experiments, we visually confirmed the presence and quantified co-expression of sgRNAs and Cas9 within TRN by identifying fluorescent markers of sgRNAs (mCherry) and Cas9 (GFP). GFP+ neurons were identified by green fluorescence in the cytoplasm (excitation:emission 488:509) and mCherry+ neurons were identified by red fluorescence in the cytoplasm (excitation:emission 590:617).

For measures of transduction success, we calculated percentages of targets based on an average of two sections per brain. mCherry signals marking AAV transduction were consistently found within the anterior-posterior location, Bregma −0.8 ± 0.2 mm. Target areas were measured using the Fiji free-hand tool (https://imagej.net/Fiji) which reports a manually drawn area in pixels. We first drew an area around the GFP+ TRN region; within this, we drew a second area which was mCherry+. Percentages reported represent the percent of the mCherry+ area divided by the total GFP+ area. Targeted cells were counted manually using the Fiji multipoint tool. We first counted all the GFP+ cells within the TRN region, then we counted all the cells that were both mCherry+ and GFP+ within that region. Percentages represent the number of double labeled (mCherry+GFP+) cells divided by total number of GFP+ cells within the TRN. Mice were considered successful cases if any TRN region was found to be positive for both mCherry and GFP. All successful cases were used to quantify transduction, as described above. In three cases, no signal for mCherry was found within the GFP+ TRN region and were excluded from the cohort, in one mouse transduction efficiency was 63.5% (area) and 66.4% (cells). The in vivo data from these four mice were used only in the serendipitous control cohort of Supplementary Fig. S4.

### In vitro slice electrophysiology

Adult (3–8 Month) mice were deeply anesthetized with isoflurane and decapitated at ZT4-5. To knockdown α3 subunits in TRN, mice were injected at 2.5 Months and slices were prepared 4–5 weeks later. Coronal brain sections containing TRN (Bregma −0.46 to −0.94 mm) were cut at 300 μm-thickness with a Leica VT1200S vibratome (Leica Biosystems Inc., Buffalo Grove, IL, USA) at 4 °C. After slicing, the slices were placed into ACSF containing the following (in mM): 124 NaCl, 1.8 KCl, 25.6 NaHCO_3_, 1.2 KH_2_PO_4_, 2 CaCl_2_, 1.3 MgSO_4_, and 10 glucose (300 mOsm), saturated with 95% O_2_/5% CO_2_ for at least 1 h at room temperature before being transferred to the recording chamber and superfused with warm ACSF (32 °C) at 2–3 ml/min.

Spontaneous inhibitory post-synaptic current (sIPSC) recordings focused on the anterior-dorsal section of the TRN and target cells were identified by GFP expression and mCherry expression. We filled 3–6 MΩ patch pipettes with intracellular solution of the following composition (in mM): 130 KCl, 5 NaCl, 2 MgCl_2_, 10 HEPES, 0.1 EGTA, 2 NA_2_ATP, 0.5 NaGTP, 4 MgATP and 1 spermine, pH 7.25 with KOH (280 mOsm). sIPSCs were recorded at −70 mV in the presence of the glutamate receptor antagonists (20 μm 6-cyano-7-nitroquinoxaline-2,3-dione +50 µM D-(2 R)-amino-5- phosphonopentanoic acid) using a Multiclamp 700B amplifier and pClamp 10.0 software (Molecular devices; California, United States). A 1 min period after 5 min application of the glutamate receptor antagonists was used for statistical analysis. Only well resolved events with amplitudes >10 pA were analyzed (Igor Pro6.02A, WaveMetrics, Inc., Portland, OR, USA). To analyze the decay of sIPSCs, in clampfit 10.2 software (Molecular Devices, LLC, San Jose, CA, USA), each sIPSCs event was detected and normalized according to its negative peak (range of 0.3 ms around the peak). An averaged trace for each cell was then generated by averaging the normalized events. The peak-to-baseline phase of the averaged trace was fitted with a two-term exponential function in GraphPad prism5 (GraphPad Software, San Diego, CA, USA). Series resistance was 6–20 MΩ and was not compensated. Sampling rate was 20 kHz. Records were low-pass (Bessel) filtered at 1 KHz.

### Statistics

We used a Jarque–Bera test to evaluate data distribution before choosing an appropriate inferential test. Normality was never violated and parametric tests were used uniformly. Multiple two-tailed paired *t*-tests were used to compare BL vs KD areas under delta power or other defined frequency bands (represented by bold black lines and symbols δ, θ or σ; ‘significant’ or ‘not significant’ is indicated by * or *n.s*. respectively) in NREM, wake or REM. We used Hommel corrected *p*-values to account for family-wise error rates to report significance. We found a unidirectional effect on NREM delta, so we used a one-tailed test in subsequent delta power analyses, comparing areas under dynamic changes in delta power at state-transitions (also marked by a bold black line with ‘significant’ or ‘not significant’ indicated by * or *n.s*. respectively). We used a two-tailed paired *t*-test in bout analyses and spindle analyses, and an unpaired *t*-test to compare control vs KD sIPSCs. All statistics were performed in MATLAB, GraphPad Prism5 or Microsoft Excel.

## Supplementary information

Supplementary information

## Data Availability

Raw in vivo electrophysiologic data^60^ used in this study are available from the Open Science Framework repository at https://osf.io/uf2ca/. Raw in vitro electrophysiologic data^61^ used in this study are available from the Open Science Framework repository at https://osf.io/2k8s3/. A Source Data file is provided with this paper.

## Code availability

Custom code central and original to this manuscript are available from the Open Science Framework repository at https://osf.io/uf2ca/.
