# Supplementary material for: Knockdown of GABAA alpha3 subunits on thalamic reticular neurons enhances deep sleep in mice
Source: Nat Commun. 2022 Apr 26;13:2246. doi: 10.1038/s41467-022-29852-x (PMC9042958; doi:10.1038/s41467-022-29852-x)
Supplement: Supplementary file 1 — Supplementary information [file 41467_2022_29852_MOESM1_ESM.pdf]

Supplementary information of:

Knockdown of GABAA alpha3 subunits on thalamic reticular neurons enhances deep sleep in mice

Authors: David S. Uygun[1], Chun Yang[1], Elena R. Tilli[2], Fumi Katsuki[1], Erik L. Hodges[1], James T. McKenna[1], James M. McNally[1], Ritchie E. Brown[1]†, Radhika Basheer[1*]†.

Affiliations:

[1]VA Boston Healthcare System and Harvard Medical School, Dept. of Psychiatry, West Roxbury, MA 02132, USA.

[2]Stonehill College, Department of Psychology, Easton, MA 02357, USA

*Correspondence to: radhika_basheer@hms.harvard.edu

† These authors jointly supervised this work

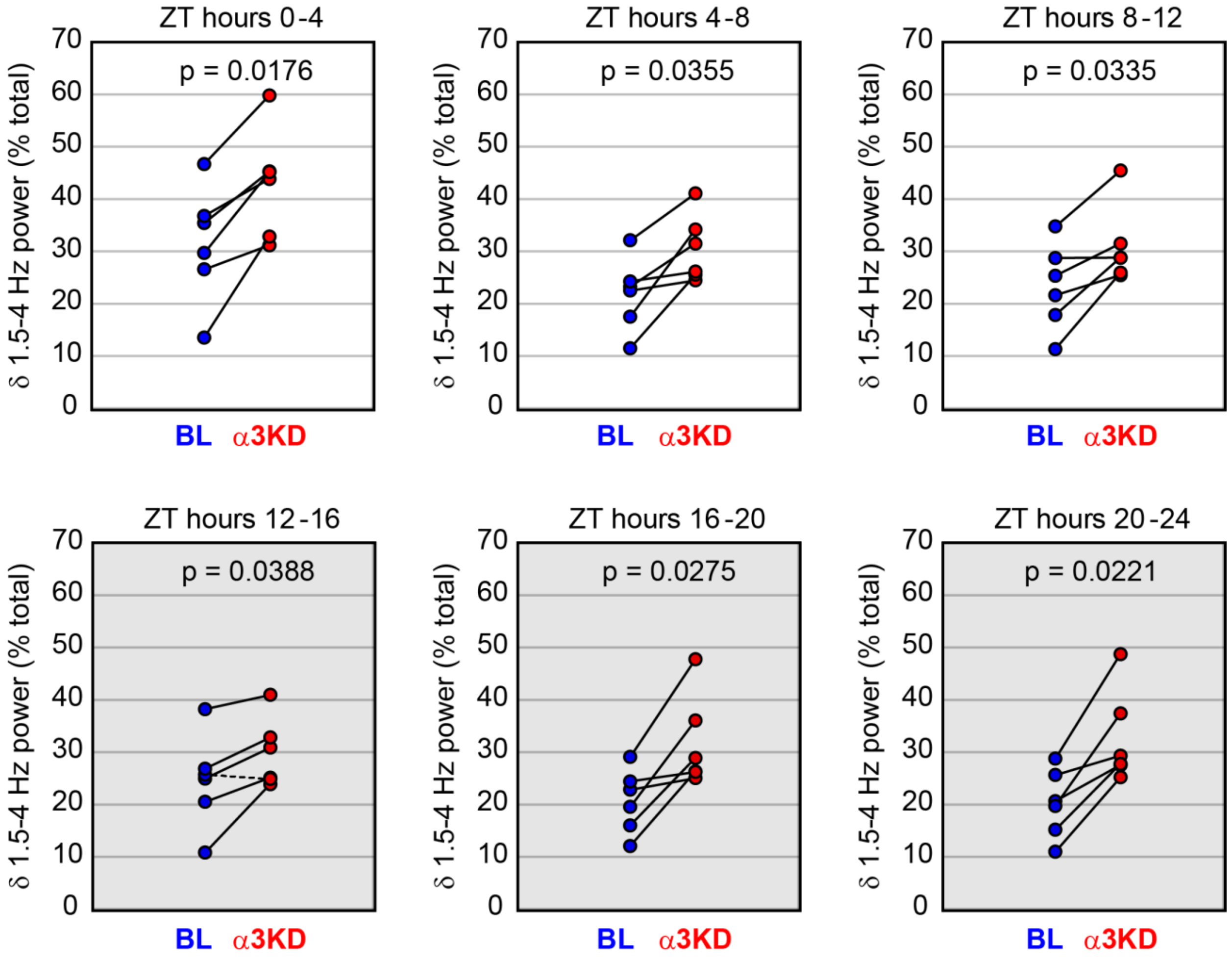

Supplementary Fig. 1. α3KD in PV+TRN neurons consistently increased NREM delta (δ, 1.5-4 Hz) power, with the effect being most prominent in the first 4 hours of the light period. Compared with their baseline (BL; blue) recordings, each α3KD (red) mouse had higher NREM delta power in every four-hour zeitgeber time (ZT) period, except in one mouse (dashed line) in the first four hours of the dark period (grey) when mice are mostly awake. Significance was determined using two-tailed paired t-tests with Hommel corrected p-values to account for family-wise error rate. N = 6.

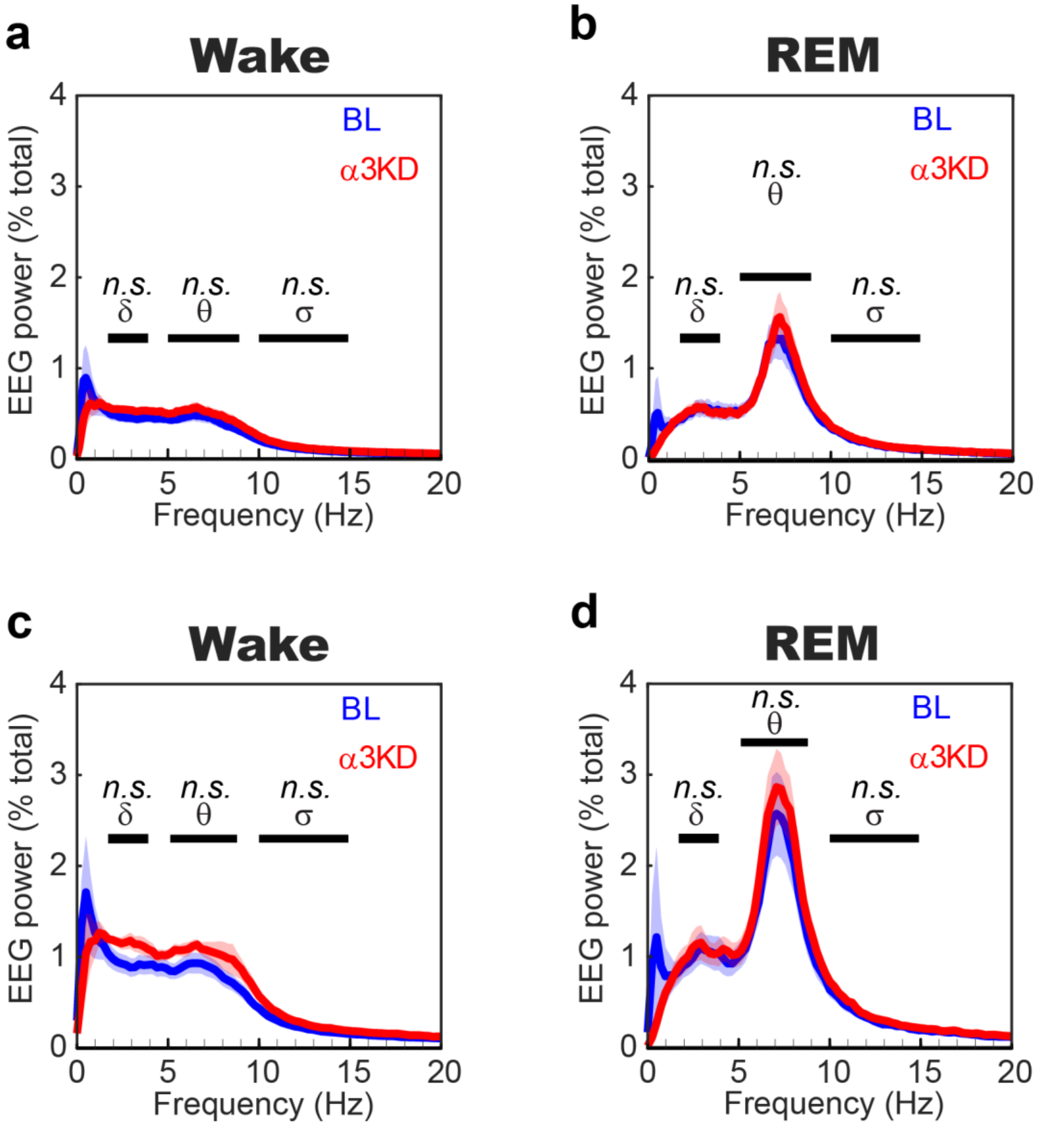
a
Wake
b
REM
c
Wake
d
REM
BL
α3KD
n.s.
δ
n.s.
θ
n.s.
σ
EEG power (% total)
Frequency (Hz)
0
1
2
3
4
0
5
10
15
20

Supplementary Fig. 2. α3KD in PV+TRN neurons did not alter the electroencephalographic (EEG) spectral profile of wakefulness or REM sleep. A. During the whole 12-hours light period, compared with their baseline (BL; blue) levels from before the α3KD was initiated, α3KD (red) mice (N=6) had the same amounts of delta (δ, 1.5-4 Hz) power, theta (θ, 5-9 Hz) power and sigma (σ, 10-15 Hz) power in wakefulness. Thick lines indicate mean; envelopes indicate SEM. b. Compared with their baseline (BL) levels (blue) from before the α3KD was initiated, α3KD (red) mice had the same amounts of delta (δ) power, theta (θ) power and sigma (σ) power in REM sleep during the light period. Thick lines indicate mean; envelopes indicate SEM. c. during the first four hours of the light period, compared with their baseline (BL) levels (blue) from before the α3KD was initiated, α3KD (red) mice had the same amounts of delta (δ) power, theta (θ) power and sigma (σ) power in wakefulness. Thick lines indicate mean; envelopes indicate SEM. d. Compared with their baseline (BL) levels (blue) from before the α3KD was initiated, α3KD (red) mice had the same amounts of delta (δ) power, theta (θ) power and sigma (σ) power in REM sleep during the first four hours of the light period. Significance was determined using two-tailed paired t-tests. Thick lines indicate mean; envelopes indicate SEM. n.s. indicates not significant.

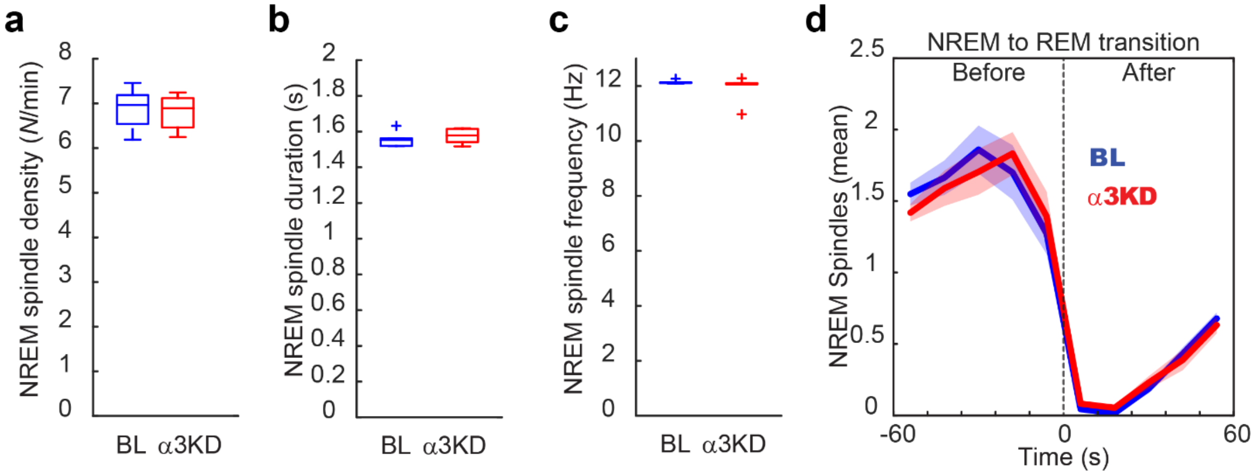

Supplementary Fig. 3. Knockdown of α3 containing $GABA_ARs$ in PV+ TRN neurons did not affect sleep spindle activity. a. Compared to baseline (BL; blue), α3KD (red; N = 6) did not change the density of NREM spindles, as measured by the number of spindles per minute of NREM ($t\ (5) = 1.27$, $p = 0.26$). b. α3KD (N = 6) did not change the duration of NREM spindles ($t\ (5) = -1.66$, $p = 0.16$). c. α3KD (N=6) did not change the frequency of NREM spindles ($t\ (5) = 1.11$, $p = 0.32$). d. α3KD did not change the number of NREM spindles within the period of NREM before a transition to REM, when sigma power and spindles surge in mice. Significance was determined using two-tailed paired t-tests. Thick lines indicate mean; envelopes indicate SEM. Box plots: center lines represent median values, 75th percentiles are box tops and 25th percentiles are box bottoms. + symbols represent outliers defined as > 1.5x the interquartile range away from box tops or bottoms.

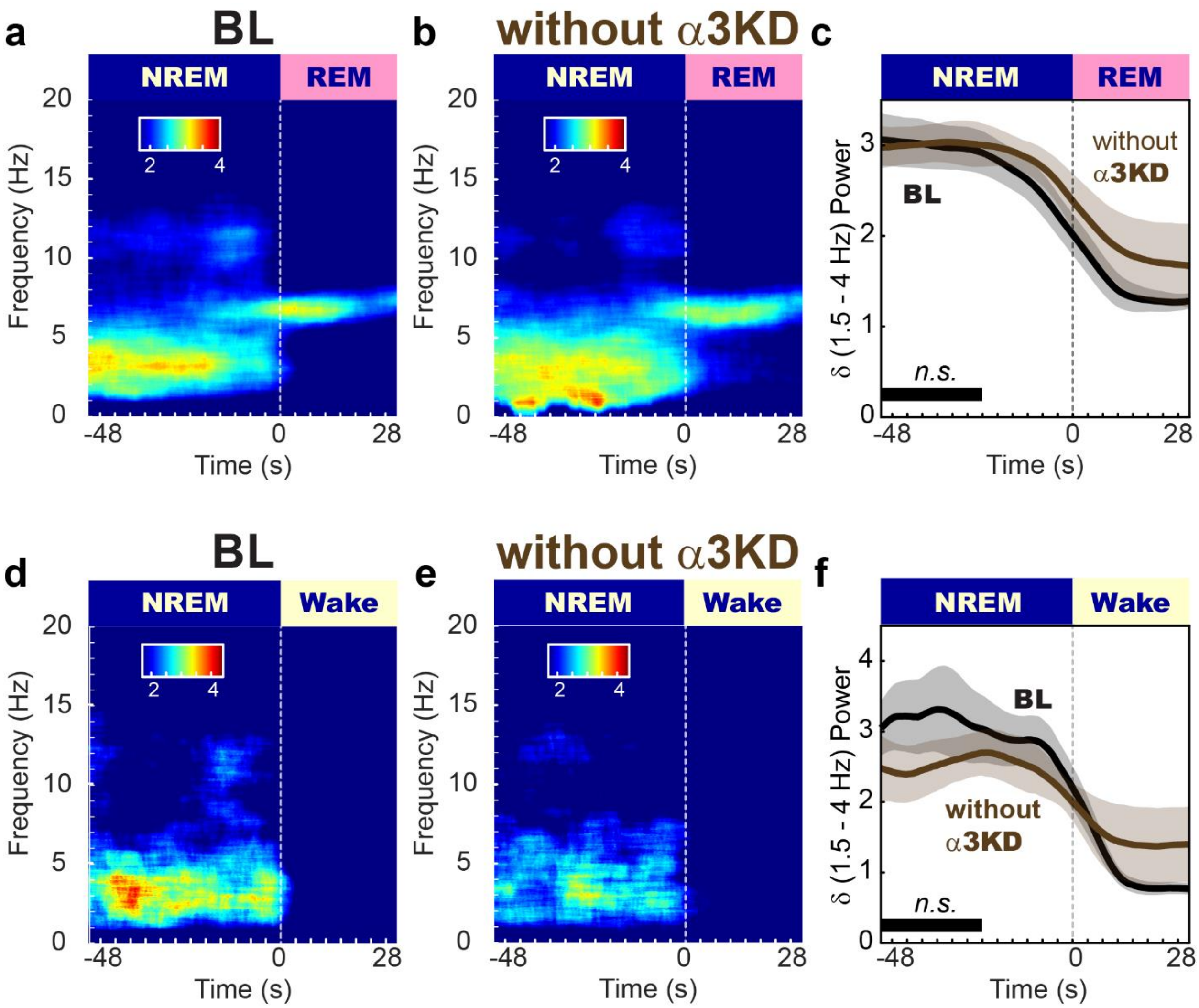

a
BL
b
without α3KD
c
NREM
REM
20
15
10
5
0
Frequency (Hz)
2
4
-48
0
28
Time (s)
δ (1.5 - 4 Hz) Power
without
α3KD
BL
n.s.
d
BL
e
without α3KD
f
NREM
Wake
without
α3KD

Supplementary Fig. 4. The control cohort with insufficient transduction of PV+TRN neurons, displayed no changes to NREM or wake time, or delta power in any states, including transitions from NREM to REM. a. Baseline (BL) time-frequency power dynamics reveals high delta power in non-rapid eye movement sleep (NREM) leading to a transition to rapid eye movement sleep (REM). b. In the control cohort with no or low transduction of PV+TRN neurons (N = 4), the delta power in NREM before a transition to REM was the same as in their BL records. c. Compared with baseline (black), the control cohort with insufficient transduction of PV+TRN neurons (brown) mice had unaltered delta (δ, 1.5-4 Hz) power in the NREM before a transition to REM ($t\ (3) = 0.03$ $p = 0.49$). Significance was tested using a one-tailed paired t-test. Thick lines indicate mean; envelopes indicate SEM. d. BL time-frequency power dynamics reveals delta power in NREM leading to a transition to wake. e. the control cohort with insufficient transduction of PV+TRN neurons did not have increased delta power in NREM before a transition to wake. f. Compared with baseline (black), the control cohort which lacked transduction of PV+TRN neurons (brown) mice had unchanged delta power in the NREM preceding a transition to wake ($t\ (3) = -1.68$, $p = 0.9$). Significance was tested using a one-tailed paired t-test. Thick lines indicate mean; envelopes indicate SEM. Data from whole 12 period of light. N = 4. Color scales represent normalized power (power at time/power from wakefulness).

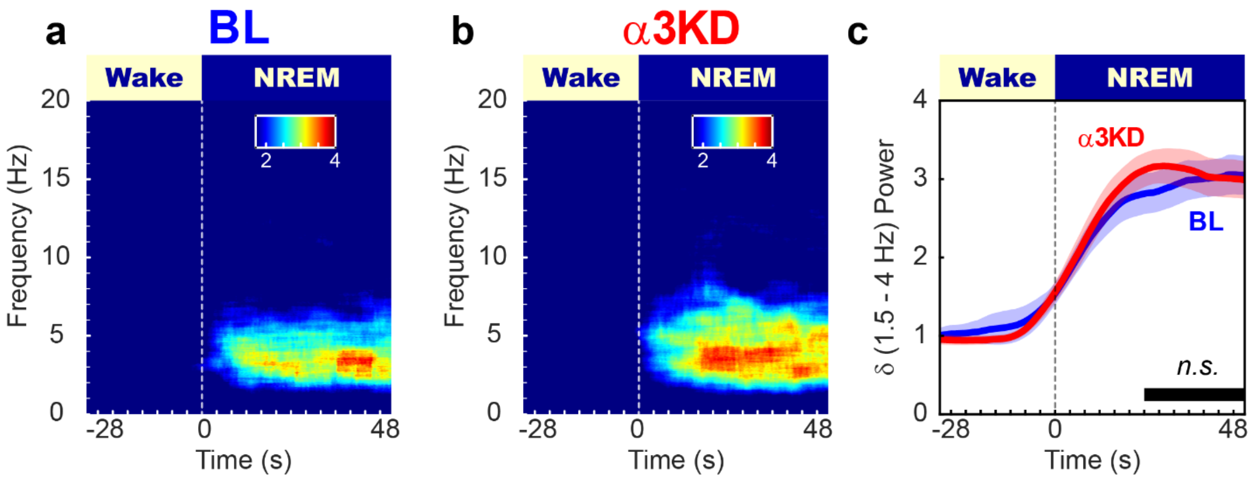

Supplementary Fig. 5. α3KD in PV+TRN neurons did not increase NREM delta power at wake to NREM transitions. a. Baseline (BL; blue) time-frequency power dynamics presents delta (δ, 1.5-4 Hz) power in non-rapid eye movement sleep (NREM) following a transition from wakefulness. b. After α3KD (red), the delta power in NREM after a transition from wakefulness appeared similar. c. Compared with their BL levels (blue), α3KD (red) mice had similar amounts of delta power in the NREM following a transition from wakefulness ($t\ (5) = 0.67$, $p = 0.27$). Data from whole 12 period of light. N = 6. Thick lines indicate mean; envelopes indicate SEM. Color scales represent normalized power (power at time/power from wakefulness). Thick lines indicate mean; envelopes indicate SEM. Color scales represent normalized power (power at time/power from wakefulness).

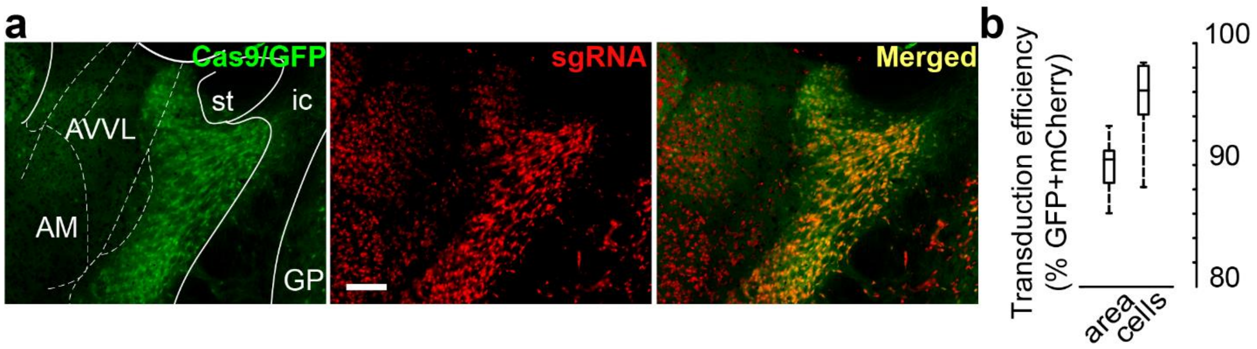

Supplementary Fig. 6. Successful targeting of PV+ TRN neurons by the control vector 'sgRNA-α1-mCherry' was validated by histology. a. GFP indicates rich Cas9 expression within the TRN region (green), mCherry reveals widespread transduction of the TRN region by the AAV vector delivering α1 targeting sgRNAs (red) with many of the cells in the area co-expressing both markers (merged; yellow). Scale bar = 200 μm. b. Percentages of target cells and target area that co-express markers reveal widespread delivery of α1 targeting sgRNAs to target cells in the mice used for *in vivo* control experiments. Box plots: center lines represent median values, 75th percentiles are box tops and 25th percentiles are box bottoms. + symbols represent outliers defined as > 1.5x the interquartile range away from box tops or bottoms. N = 7. [Adapted from Franklin and Paxinos[59], with permission from Elsevier].

Frequency band	Zeitgeber period	0-4	4-8	8-12	12-16	16-20	20-24	0-12	12-24
0.5-1.5 Hz	BL mean	8.8	8.5	8.2	9.4	8.8	9.1	7.7	8.8
	BL SEM	1.7	0.9	1.0	1.8	1.0	1.5	1.5	1.8
	KD mean	11.1	8.9	8.9	8.9	9.2	9.2	9.1	7.5
	KD SEM	0.9	0.6	0.8	0.6	0.9	0.8	0.4	0.6
	P value	0.1550	0.6812	0.5608	0.8100	0.7392	0.9273	0.4363	0.448
0-1 Hz	BL mean	5.7	9.3	8.2	9.4	10.4	12.4	4.8	9.9
	BL SEM	2.2	2.4	2.6	3.1	3.3	3.9	2.0	2.9
	KD mean	5.6	5.5	5.4	5.7	5.5	5.4	4.0	4.3
	KD SEM	0.8	1.0	1.0	1.1	1.2	0.9	0.6	0.9
	P value	0.9563	0.1700	0.3521	0.3377	0.2210	0.1117	0.7677	0.084
0.5-1.75 Hz	BL mean	11.6	10.7	10.4	11.8	10.9	11.2	10.3	10.9
	BL SEM	2.1	1.0	1.2	2.0	1.1	1.6	1.9	1.9
	KD mean	15.1	11.8	11.9	11.8	12.3	12.3	12.4	9.9
	KD SEM	1.2	0.7	1.0	0.8	1.2	1.1	0.5	0.6
	P value	0.0685	0.2678	0.3006	0.9955	0.3845	0.5263	0.3137	0.599
0.75-1.75 Hz "δ1"	BL mean	10.0	8.5	8.4	9.4	8.4	8.3	9.0	8.8
	BL SEM	1.7	0.9	1.0	1.5	0.7	1.0	1.5	1.4
	KD mean	13.4	10.2	10.3	10.1	10.6	10.6	11.2	8.5
	KD SEM	1.2	0.6	0.9	0.7	1.1	1.1	0.5	0.4
	P value	0.0125	0.0228	0.0367	0.5881	0.0432	0.0550	0.1337	0.823
1.5-4 Hz	BL mean	31.4	21.8	23.3	24.5	20.6	20.1	29.9	20.3
	BL SEM	4.6	2.8	3.4	3.6	2.5	2.7	4.4	2.5
	KD mean	43.0	30.5	31.0	29.7	31.7	32.7	37.7	25.7
	KD SEM	4.2	2.6	3.0	2.7	3.6	3.6	2.6	1.6
	P value	0.0035	0.0178	0.0168	0.0388	0.0138	0.0044	0.0395	0.020
2-4.5 Hz	BL mean	31.2	21.4	22.9	24.1	20.3	19.7	30.0	20.1
	BL SEM	4.3	2.8	3.3	3.5	2.6	2.7	4.2	2.4
	KD mean	42.0	29.7	30.2	29.0	30.8	32.0	37.6	25.5
	KD SEM	3.8	2.4	2.7	2.5	3.3	3.3	2.4	1.6
	P value	0.0073	0.0301	0.0263	0.0607	0.0192	0.0045	0.0507	0.023
2.5-3.5 Hz "δ2"	BL mean	13.2	9.0	9.6	10.1	8.4	8.2	12.5	8.2
	BL SEM	1.9	1.2	1.5	1.5	1.1	1.2	1.8	1.1
	KD mean	17.9	12.6	12.8	12.2	13.2	13.5	15.8	10.6
	KD SEM	1.7	1.1	1.2	1.1	1.5	1.4	1.1	0.7
	P value	0.0048	0.0259	0.0230	0.0557	0.0178	0.0045	0.044	0.023

Supplementary Table 1. Comparison of power in delta and slow waves with diverse defining band-widths. α3 KD increased the power in various delta bands but not in slow wave bands. P values are from two-tailed paired t-tests. Significant p-values reported in red. Hommel corrected p values for the 1.5-4 Hz band are given in Extended Data Fig 1.

	Area (pixels)							Cells (N)						
N	Anterior to injection site (AP-0.6-0.7mm)			Posterior to injection site (AP -0.7-0.9mm)			% (mean)	Anterior to injection site (AP -0.6-0.7mm)			Posterior to injection site (AP-0.7-0.9mm)			% (mean)
	Green	Red	%	Green	Red	%		Green	Red	%	Green	Red	%	
1	127485	123800	97.1	185060	179433	97.0	97.0	248	247	99.6	313	308	98.4	99.0
2	340910	305723	89.7	332366	312618	94.1	91.9	374	324	86.6	171	144	84.2	85.4
3	75462	63648	84.3	168074	164505	97.9	91.1	146	136	93.2	199	179	89.9	91.6
4	585220	565528	96.6	408257	375366	91.9	94.3	631	618	97.9	330	305	92.4	95.2
5	566760	546778	96.5	471721	459172	97.3	96.9	585	565	96.6	346	340	98.3	97.4
6	355778	340242	95.6	617168	584501	94.7	95.2	323	301	93.2	622	596	95.8	94.5

Supplementary Table 2. Quantification of viral transduction confirmed high efficiency. α3KD percentage areas (pixels) and cells (N) measuring transduction efficiency within target neurons of the TRN as measured by mCherry+ (Red) within the GFP+ (Green) TRN region over total GFP+ TRN region.

Off target labelling

Mouse	TC relay (Medial spread) mCherry+ cells (N)	TC relay (Medial spread) mCherry+GFP+ cells (N)	TC relay (Medial spread) %	Globus Pallidus (Lateral spread) mCherry+ cells (N)	Globus Pallidus (Lateral spread) mCherry+GFP+ cells (N)	Globus Pallidus (Lateral spread) %
1	555	16	2.9	179	0	0.0
2	96	0	0.0	128	11	8.6
3	0	0	N/A	0	0	N/A
4	744	94	12.6	193	3	1.6
5	362	29	8.0	314	16	5.1
6	495	7	1.4	438	18	4.1
Mean			5.0			3.9
SD			5.2			3.3

Supplementary Table 3. Quantification of viral transduction outside the TRN revealed low levels of off-target medial and lateral labeling restricted to areas devoid of $\alpha3$ subunits i.e. TC relay nuclei and globus pallidus.
